# Role of *myo*-inositol during skotomorphogenesis in *Arabidopsis*

**DOI:** 10.1038/s41598-020-73677-x

**Published:** 2020-10-15

**Authors:** Naveen Sharma, Chanderkant Chaudhary, Paramjit Khurana

**Affiliations:** Department of Plant Molecular Biology, University of Delhi South Campus, Benito Juarez Road, New Delhi, 110021 India

**Keywords:** Developmental biology, Molecular biology, Plant sciences

## Abstract

*Myo*-inositol is a ubiquitous metabolite of plants. It is synthesized by a highly conserved enzyme L-*myo*-inositol phosphate synthase (MIPS; EC 5.5.1.4). *Myo*-inositol is well characterized during abiotic stress tolerance but its role during growth and development is unclear. In this study, we demonstrate that the apical hook maintenance and hypocotyl growth depend on *myo*-inositol. We discovered the *myo*-inositol role during hook formation and its maintenance via ethylene pathway in *Arabidopsis* by supplementation assays and qPCR. Our results suggest an essential requirement of *myo*-inositol for mediating the ethylene response and its interaction with brassinosteroid to regulate the skotomorphogenesis. A model is proposed outlining how *MIPS* regulates apical hook formation and hypocotyl growth.

## Introduction

The apical hook formation and maintenance is one of crucial developmental process in higher plants as it protects the shoot apical meristem (SAM) till seedling emergence out of the soil^12^. Apical hook formation occurs soon after germination and is maintained while seedlings makes their way through the soil and terminates upon exposure to light. The apical hook formation is orchestrated by a variety of hormones which leads to differential cell elongation in the hypocotyl^1^. Apical hook formation goes through three consecutive growth phases i.e., formation, maintenance and opening^41^. The plant hormones, auxin and ethylene interaction leads to differential growth in the formation of apical hook^34^. They have been involved in differential growth in the apical hook^19,34^. However, the mechanism by which ethylene triggers differential growth in the hypocotyl still far from understood.

Ethylene enhances apical hook curvature as observed upon application of exogenous ethylene^17,19^ in constitutive triple response1 (*ctr1*) mutant^30^ and the ethylene overproducer (*eto*) mutants^55^. Auxin and ethylene are involved in differential growth in apical hook, in which auxin results in cell expansion and hypostyle growth, while ethylene has an antagonistic effect^34^. Moreover, ethylene has a stimulatory effect on the auxin biosynthetic pathway^51^ which suggests another mode of interaction at the hormone level. As both auxin and ethylene are involved in the regulation of apical hook development, their activities are mutually coordinated.

Auxin gradient is one of critical factor for apical hook formation^1,35^. *Arabidopsis* mutants of auxin response such as TRANSPORT INHIBITOR RESPONSE 1 (TIR1) and AFB members^15^ and over-accumulating free auxin^8,34^, exhibit hookless phenotype. Exogenous treatment with auxin and of polar auxin transport inhibitors affects hook curvature^34,49^, which suggests that optimal auxin transport is critical for differential growth in the apical hook and is regulated by dedicated influx and efflux carriers like AUX/LAX family of auxin influx carriers^1,56^, the PIN family of auxin efflux carriers^1,38^ and by the B-type ATP-binding cassette transporters (ABC transporters)^45^.

Brassinosteroid (BR) is a steroid hormone which binds to receptor kinase BRI1 to initiate the signal transduction, e.g. inactivation of GSK3-like kinase; BIN2, dephosphorylation of BRASSINAZOLE RESISTANT 1 (BZR1) family transcription factor; accumulation of unphosphorylated BZR1 in the nucleus; and regulation of BR target genes^9^. BR mutants defective in BR synthesis like *det2*, *cbb1*, *cpd* shows no hook formation during skotomorphogenesis^10,29,52^ and at lower concentration BR stimulates stem growth^21^. Exogenous ethylene treatment results in altered auxin gradient either directly or change in BR biosynthesis^19^. All the three hormones, BR, ethylene and auxin affect each other and are necessary for apical hook formation.

Previous reports showed that MIPS has been involved in cell wall biogenesis^36^, auxin storage^2^, phytic acid synthesis^3^ and oligosaccharides synthesis^28^. MIPS is also required for PIN protein localization, polar auxin transport and auxin-regulated embryogenesis^11,37^. Its over-expression results in resistance towards cold, drought and salt stress in several plants along with immunity towards stem nematodes in transgenic sweet potato^27,32,41,46,53,54,59^. Programmed cell death (PCD) was observed in *AtMIPS1* mutant which is light dependent and results in enhanced basal immunity^16,43^. Interestingly, Ma and coworkers in 2016 indicated the role of light signaling protein i.e. FHY3 and FAR1 in maintenance of optimal level of *myo*-inositol via directly binding to the promoter of MIPS1 and activating its expression^39^. Recent study of MIPS has revealed its critical role in growth and immunity via ethylene^50^.

Our investigation on etiolated seedlings of *Arabidopsis* show that apical hook and hypocotyl development also depends on the *myo*-inositol level. *Myo*-inositol induced hook maintenance and subsequent stimulation of ethylene biosynthetic genes and auxin transporters suggest that the ethylene effect might be mediated by *myo*-inositol. Furthermore, *myo*-inositol (MI) antagonizes brassinosteroid (BR) effects during hook formation which demonstrate an important step of regulation of BR-mediated growth and development. Thus, we conclude that differential MIPS levels are required for optimal hook formation, maintenance and hypocotyl growth.

## Results

### *Myo*-inositol maintains the apical hook

**Figure 1:**
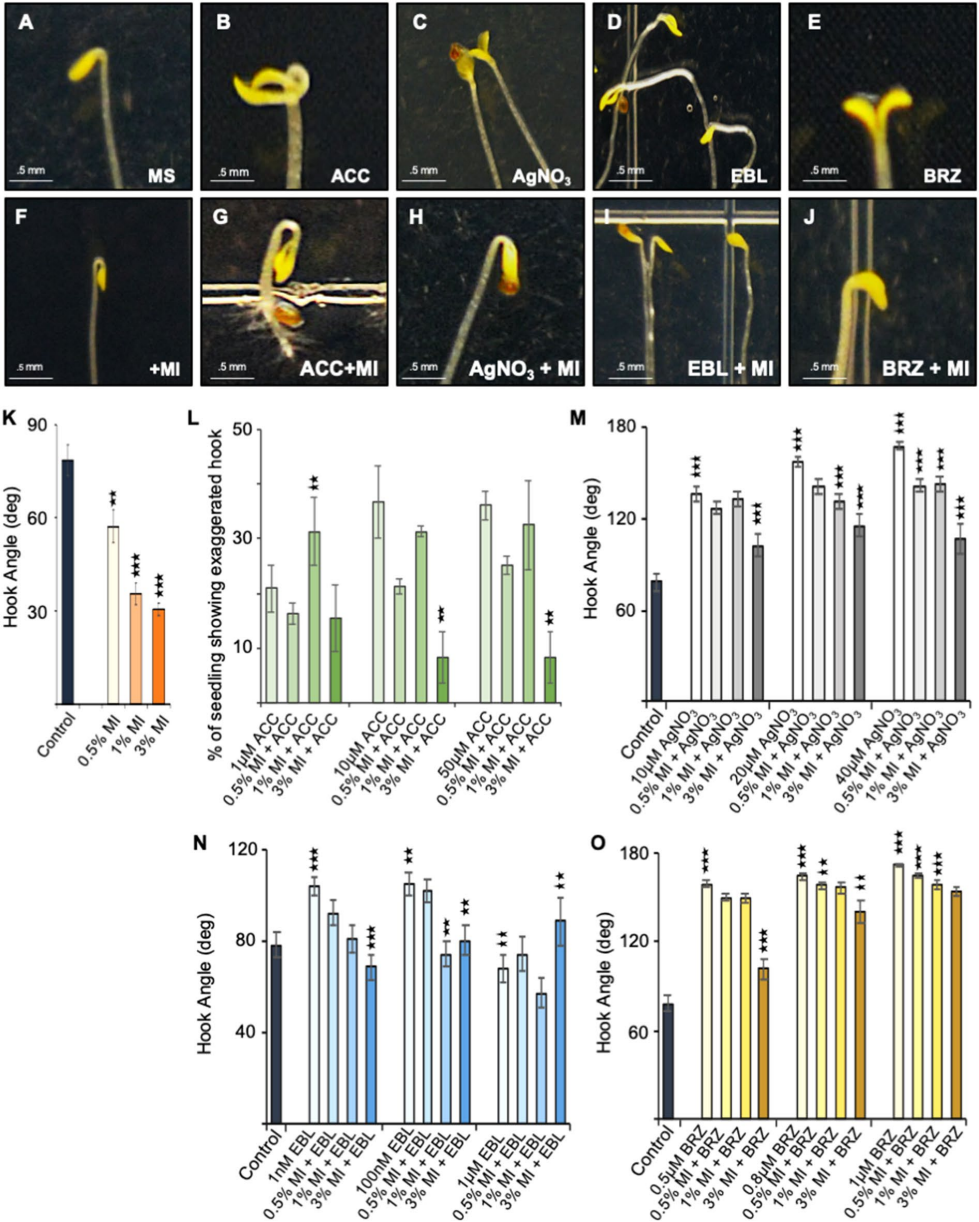
Effect of myo-inositol (MI), ethylene (ACC), AgNO_3_ brassinosteroid and brassinazole (BRZ) on hook formation and maintenance in etiolated seedling. (**A**–**E**) Photograph of shoot apices of 5-day-old wild-type etiolated seedlings grown, where indicated, on ½ MS, 1 μM ACC, 20 μM AgNO_3_, 100 nM EBL, 0.8 μM BRZ-containing media and (**F**–**J**) in combination of MI. (**K**) Apical hook angle of 5-day-old etiolated seedlings on three different concentration of MI. (**L**) Exaggerated apical hook phenotype of 5-day-old etiolated seedlings upon three concentration of ACC and with combination with MI. (**M**–**O**) Apical hook angle of 5-day-old etiolated seedlings upon three concentration of AgNO_3_, EBL, BRZ and combination of these with three MI concentration. Hook angle is measured using ImageJ, 1.8.0_172, https://imagej.nih.gov/ij/. Data shown is the average of two representative biological replicates having at least 20 seedlings; error bars represent SE. Statistical differences between control and each treatment were analyzed using Student’s t test with paired two-tailed distribution: ***P < 0.001 and **P < 0.01.

**Figure 2:**
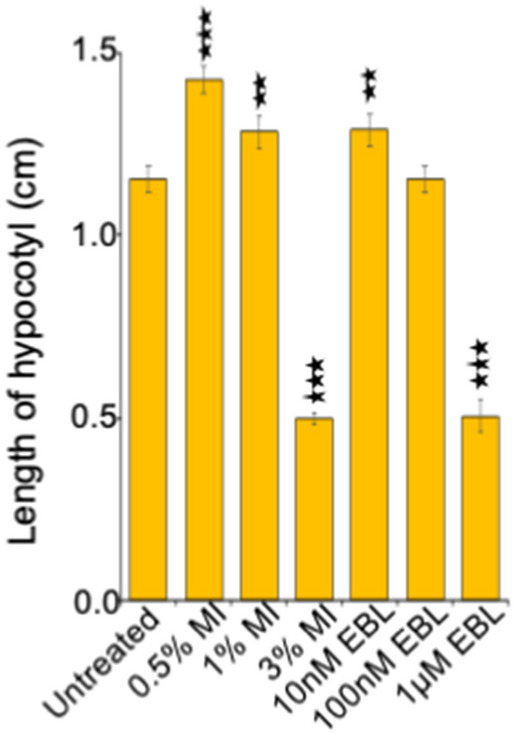
Hypocotyl length of 5-day-old etiolated seedlings upon three concentrations of MI and EBL. Hook angle is measured using ImageJ, 1.8.0_172, https://imagej.nih.gov/ij/. Data shown is the average of two representative biological replicates having at least 20 seedlings; error bars represent SE. Statistical differences between control and each treatment were analyzed using Student’s t test with paired two-tailed distribution: ***P < 0.001 and **P < 0.01.

**Figure 3:**
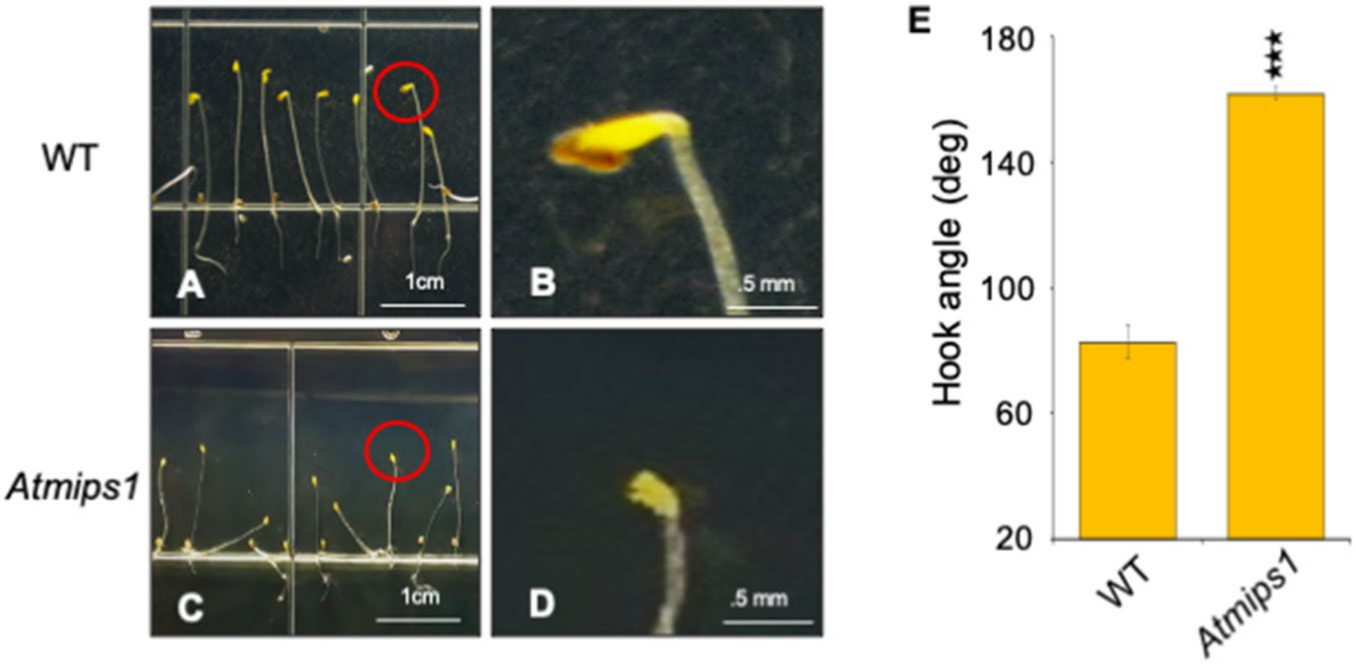
(**A**) WT 5-day old etiolated seedling grown on ½ MS media without chemical treatment. (**C**) *Atmips1* mutant 5-day old etiolated seedling grown on ½ MS media without chemical treatment. (**B**,**D**) Enlarged view of selected area. Red coloured circle depicts the selected area. (**E**) Apical hook angle of 5-day-old etiolated seedlings (WT, *Atmips1* mutant). Hook angle is measured using ImageJ, 1.8.0_172, https://imagej.nih.gov/ij/. Data shown is the average of two representative biological replicates having at least 20 seedlings; error bars represent SE. Statistical differences between control and each treatment were analyzed using Student’s t test with paired two-tailed distribution: ***P < 0.001 and **P < 0.01.

Our previous study with *myo*-inositol phosphate synthase (MIPS) suggest its role in ethylene response^50^. To further assess the role of *myo*-inositol (MI) in ethylene response, we analyzed the effect of *myo*-inositol on etiolated seedlings of *Arabidopsis* Col-0. Exogenous MI supplementation resulted in decrease in length of the hypocotyls and hook angle (Figs. 1F, 2) compared to control condition (Fig. 1A). We analysed three concentrations of MI and found significant decrease in magnitude of the hook angle from 0.5 to 1% MI (Fig. 1K; Supplementary Figure S1). We also investigated the triple response caused by ethylene by 1-aminocyclopropane-1-carboxylic acid (ACC) supplementation (Fig. 1B) and in combination of ACC and MI (Fig. 1G). We found an increase in number of seedlings showing exaggerated apical hook formation with increasing concentration of ACC (Fig. 1L; Supplementary Figure S2). However, MI and ACC combination resulted in declination. We also analysed development of the *Atmips1* mutant during dark (Fig. 3C, and Supplementary Figure S10). We observed significant increase in hook angle in *Atmips1* mutant etiolated seedlings as compared to WT (Fig. 3A,B,E). These results suggest that *myo*-inositol role in ethylene response and hook formation.

### *Myo*-inositol acts downstream of *etr* in hook formation

To address whether MI acts on ethylene biosynthesis or signaling pathway, we germinated the *Arabidopsis* Col-0 seeds on three different concentrations of AgNO_3_ and found no hook formation at all three concentrations (Fig. 1C; Supplementary Figure S3). Further, when seeds were grown on media containing different combinations of AgNO_3_ and MI, a decrease was observed in the hook angle with an increasing MI concentration (Fig. 1H,M; Supplementary Figure S3). Surprisingly, we observed hook angle as low as 15° at 3% MI along with decrease in hypocotyl length (Fig. 1H).

### *Myo*-inositol antagonizes brassinosteroid (BR) response

Due to the established role of BR in hook formation during skotomorphogenesis, we tried to discover relationship between MI and BR and carried out combination assay. We observed peculiar phenotype in etiolated seedlings upon epibrassinolide (EBL) treatment i.e. randomized growth of hypocotyls. Maximum randomization was observed at 100 nM concentration of EBL (Fig. 1D) where MI antagonizes this effect (Fig. 1I; Supplementary Figure S4). MI supplementation resulted in reduced randomization and decrease in hook angle (Fig. 1N). Upon 1% MI supplementation, we observe the same without any effect on hypocotyl length (Fig. 1I) however, 3% MI resulted in decrease of hypocotyl length but normal hook formation (Supplementary Figure S4O). In addition, we also carried out the Brassinazole (BRZ) and MI combinatorial assay and decrease was observed in hypocotyl length and increase in hook angle along with open cotyledon with increasing concentration of BRZ (Fig. 1E; Supplementary Figure S5). When BRZ was supplemented with MI, etiolated seedlings showed decrease in hypocotyl growth and hook angle. We observed a hook angle as low as 52° hook formation at 0.8 μM BRZ with 3% MI concentration (Fig. 1J,O). Data thus suggests a direct link between the BR and MI.

### Ethylene signaling inhibitor (AgNO_3_) enhance the brassinosteroid response

To investigate the effect of ethylene signaling inhibitor on BR response, we grew *Arabidopsis* seeds on media containing a combination of EBL and AgNO_3_. We found acute randomized growth of etiolated seedlings at different combination of EBL and AgNO_3_, which was increasing with increase in EBL and AgNO_3_ levels (Supplementary Figure S6). Randomization of etiolated seedlings grown at combination of 10 nM EBL and 10 μM AgNO_3_ (Supplementary Figure S6C) was more than 100 nM concertation of EBL (Supplementary Figure S4D). Short hypocotyl and exaggerated hook formation observed at 1 μM EBL was antagonized with supplementation of AgNO_3_. Similarly, we observed more randomization of hypocotyls at different combination of EBL and LiCl (Supplementary Figure S7). We thus conclude that absence of ethylene or *myo*-inositol during skotomorphogenesis exaggerates the BR effect.

### *Myo*-inositol cannot evoke hook formation in the presence of auxin transport inhibitors

**Figure 4:**
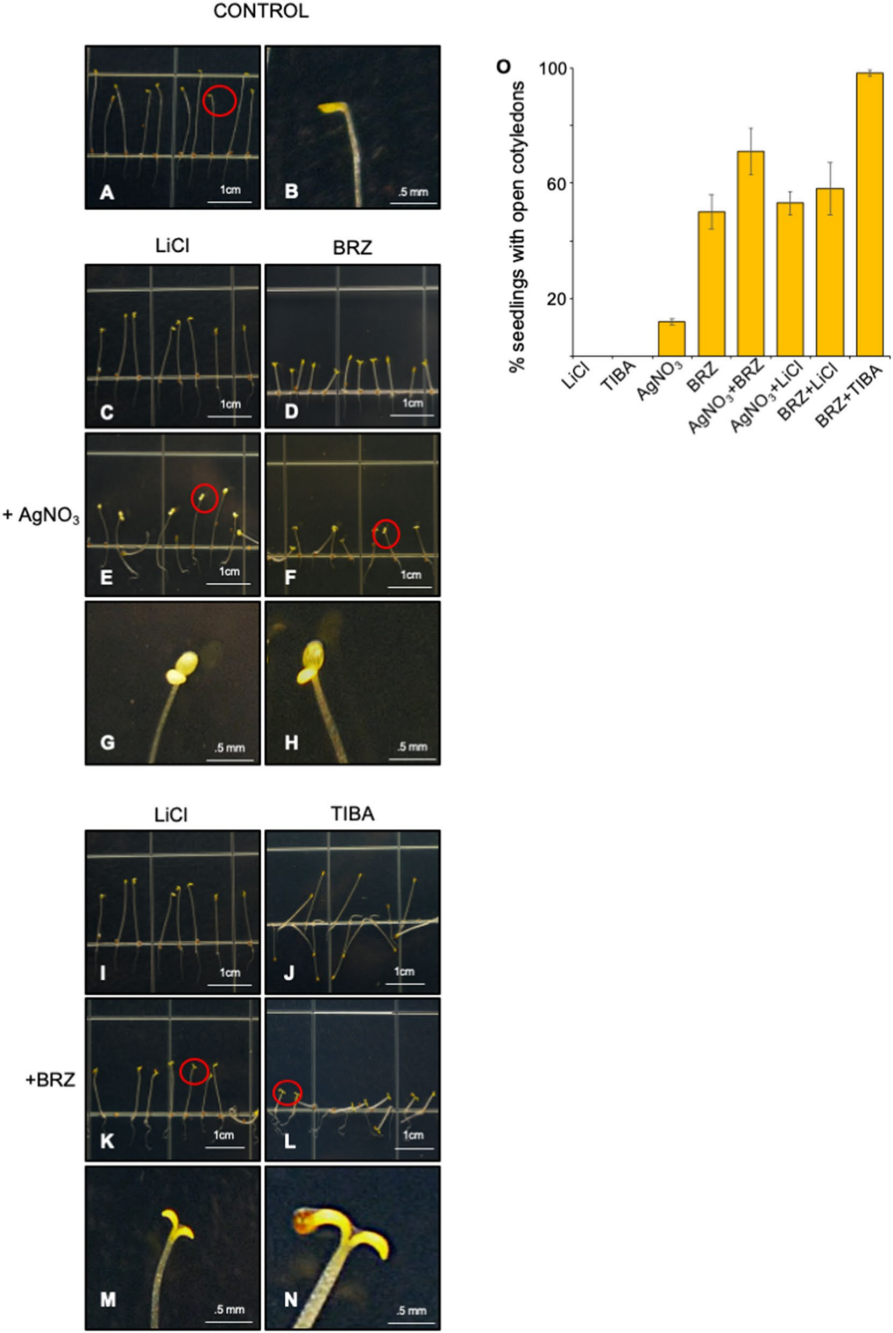
Responses of etiolated *arabidopsis* seedlings to combination of AgNO_3_, BRZ, LiCl, TIBA. (**A**) 5-day old etiolated seedling grown on ½ MS media without chemical treatment. (**C**,**D**,**I**,**J**) Etiolated *Arabidopsis* Seedlings grown on 10 mM LiCl, 0.8 μM BRZ, 10 mM LiCl, 5 μM TIBA. (**E**,**F**) Etiolated seedling grown on media containing combination 20 μM AgNO_3_ with 10 mM LiCl and 0.8 μM BRZ. (**K**,**L**). Etiolated seedling grown on media containing in combination 0.8 μM BRZ with 10 mM LiCl and 5 μM TIBA. (**B**,**G**,**H**,**M**,**N**) Enlarged view of selected area. Red coloured circle depicts the selected area. (**O**) Percentage of etiolated seedlings showing open cotyledons in combination assays with AgNO_3_, BRZ, LiCl, TIBA.

We next investigated the effect of MI in presence of IAA and auxin transporter inhibitor TIBA. We observed agravitropic growth of etiolated seedlings upon IAA treatment, however with MI supplementation results in decrease in hook angle and gravitropic growth (Supplementary Figure S8). We also checked the effect of TIBA on etiolated seedling and found no hook formation with no change in hypocotyl length (Fig. 4J; Supplementary Figure S9C–E). Upon MI supplementation, we observed no change in apical hook except a decrease in hypocotyl length with increasing MI concentration (Supplementary Figure S9). Maximum decrease in hypocotyl length was observed at 3% MI (Supplementary Figure S9O–Q).

### Skotomorphogenesis is under control of brassinosteroid, auxin and ethylene via *Myo*-inositol

To delve deeper into the role *myo*-inositol during skotomorphogenesis, we performed combination assays with inhibitors of *myo*-inositol, ethylene, brassinosteroid and auxin. When we analysed the etiolated seedlings grown on AgNO_3_ (Fig. 1C), LiCl (Fig. 4C,I), BRZ (Fig. 4D) and TIBA (Fig. 4J) with grown on MS (Fig. 4A,B). We observed an increase in apical hook angle and open cotyledon phenotype. We also analysed the etiolated seedlings grown on combination of AgNO_3_ with LiCl and BRZ. We observed more numbers of etiolated seedling showing open cotyledon phenotype (Fig. 4E–H). The percentage of open cotyledon seedlings were significantly high in AgNO_3_ and BRZ (71%) followed by AgNO_3_ and LiCl (53%) when compared to AgNO_3_ (12%), BRZ (50%) and LiCl (0%) (Fig. 4E,F,O). A decrease in hypocotyl length was observed in presence of BRZ. A similar phenotype was observed with a combination of BRZ with LiCl and TIBA (Fig. 4K–N). Percentage of etiolated seedlings showing open cotyledon phenotype which were highest with supplementation of TIBA (98%) followed by LiCl (58%) as compared to BRZ (50%), LiCl (0%) and TIBA (0%) (Fig. 4O), however BRZ could not decrease the hypocotyl length in presence of LiCl (Fig. 4K).

### Expression analysis of *MIPS* and hormone related genes in etiolated seedlings

**Figure 5:**
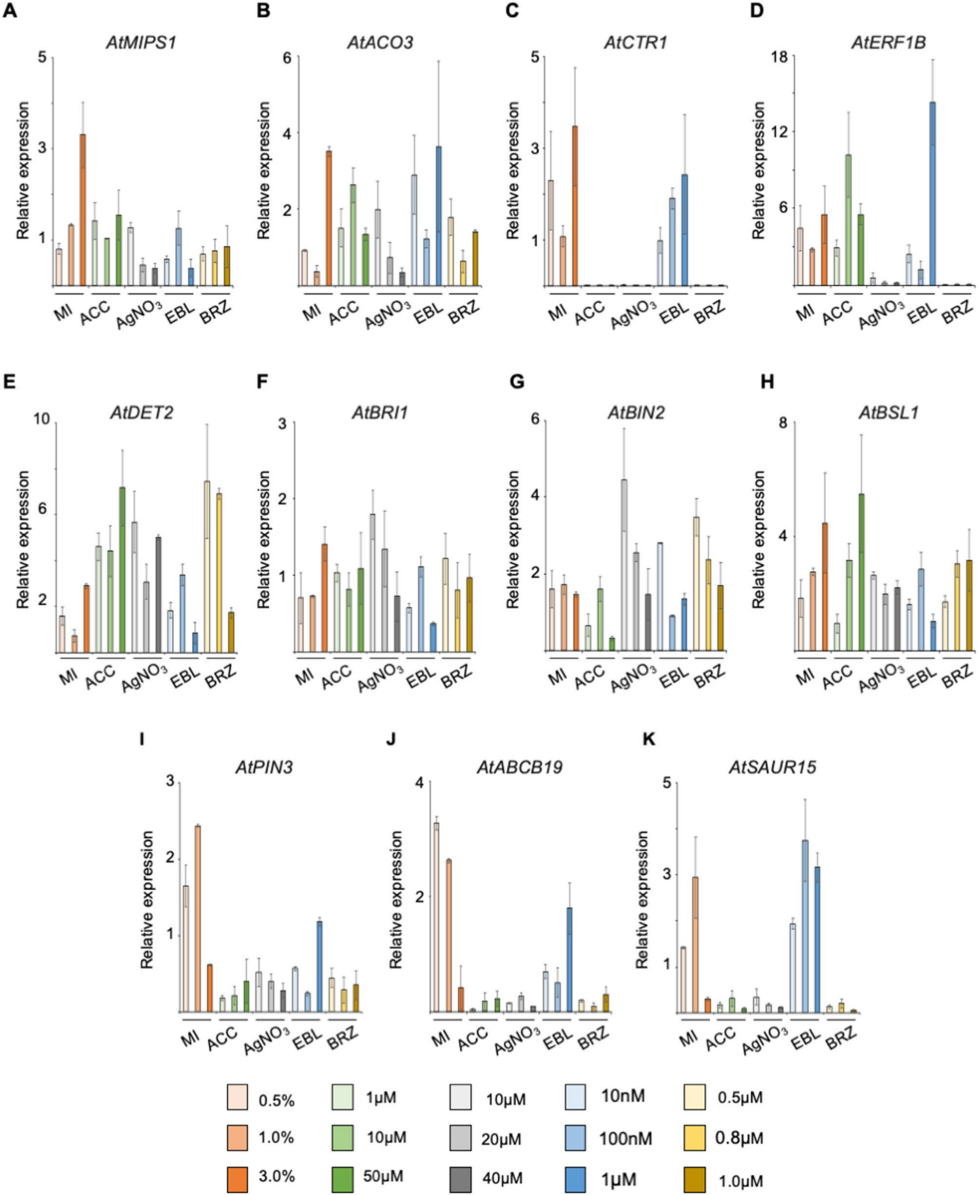
Relative expression of biosynthetic and signaling related genes of *myo*-inositol, brassinosteroid, ethylene and auxin by quantitative RT-PCR of 5-days old etiolated seedlings under different treatments. (**A–L**) Effect of three different concentration of MI, ACC, AgNO_3_, EBL and BRZ on the expression of *AtMIPS1, AtACO3, AtCTR1, AtERF1B, AtDET2, AtBRI1, AtBIN2, AtBSL1, AtBAK1, AtPIN3, AtABCB19* and *AtSAUR15* in 5-days old etiolated seedling grown on respective chemical agent. Data shown here is means $\pm$SE. Data represented are relative to *GAPDH*.

**Figure 6:**
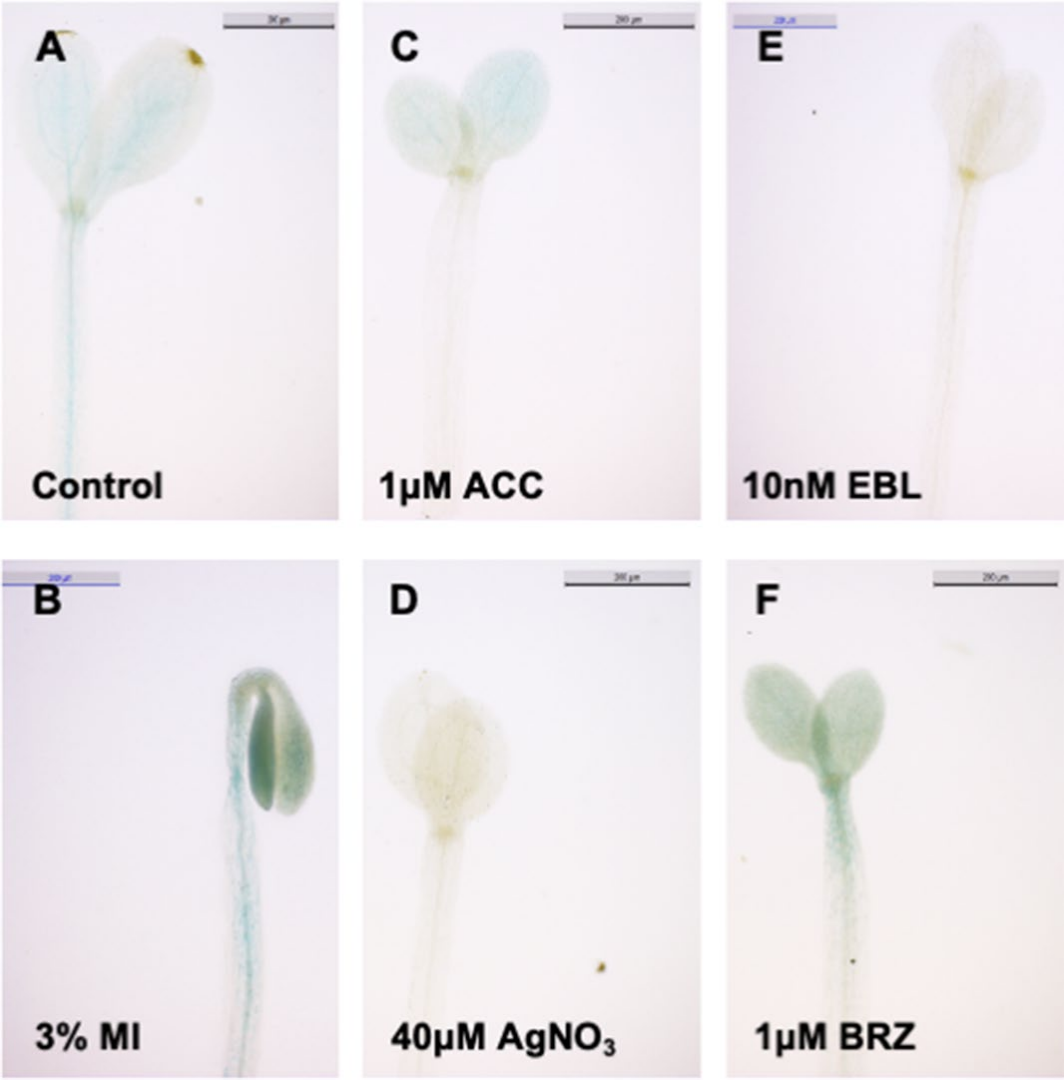
Expression pattern of MIPS1 gene.

To address whether genes involved in ethylene, brassinosteroid, auxin biosynthesis, signaling or transport are regulated by *myo*-inositol, we carried out the quantitative RT-PCR of *AtACO3* (Fig. 5B)*, AtCTR1* (Fig. 5C)*, AtERF1B* (Fig. 5D)*, AtDET2* (Fig. 5E)*, AtBRI1* (Fig. 5F)*, AtBIN2* (Fig. 5G)*, AtBSL1* (Fig. 5H)*, AtPIN3* (Fig. 5I)*, AtABCB19* (Fig. 5J)*,* and *AtSAUR15* (Fig. 5K) under various concentrations of MI, EBL, ACC, AgNO_3_ and BRZ. We also checked the expression of *myo*-inositol synthesizing enzyme gene *AtMIPS1* (Fig. 5A). An increase in expression of *AtMIPS1* was observed upon increasing concentration of MI (Fig. 5A) indicating inducible expression of *AtMIPS1* upon MI supplementation. Regulation of ethylene and brassinosteroid synthesis gene (*AtACO3, AtERF1B, AtDET2*) was observed upon MI and ACC treatment (Fig. 5B,D,E,H). However, BR treatment resulted in decrease in *AtACO3* and *AtERF1B* expression till 100 nM and a distinct increase at saturating concentration of 1 μM was observed (Fig. 5B,D). As expected, feedback mechanism of gene expression in brassinosteroid and ethylene pathways was observed (Fig. 5B,D,E,F,H) except for *AtBIN2*. An interesting pattern was observed between *AtACO3*, *AtERF1B* and *AtMIPS1* expression upon ACC treatment. We observed a bell curve in *AtACO3*, *AtERF1B* expression whereas it was an inverted bell curve in *AtMIPS1* expression (Fig. 5A,B,D). Increased expression of *AtPIN3* and *AtABCB19* were observed only in MI and 1 μM EBL with highest in MI treated etiolated seedlings (Fig. 5I,J). We also checked the expression of early auxin-inducible gene *AtSAUR15* and found its expression getting increased by the MI and EBL supplementation whereas no significant change was observed in ACC treatment (Fig. 5K). Plants expressing MIPS1 promoter fused to the *uidA* gene were also analysed. In 5-day-old etiolated seedlings of *ProMIPS1-uidA*, we observed higher β-glucuronidase (GUS) activity in MI (Fig. 6B) and BRZ (Fig. 6F) and no expression when supplemented with AgNO_3_ (Fig. 6D) and EBL (Fig. 6E) treated plants as compared to Control etiolated seedlings (Fig. 6A). Moreover, we observed more expression in cotyledon with no expressions in hypocotyl in ACC treated etiolated seedlings (Fig. 6C).

## Discussion

*Myo*-inositol has numerous roles in plants i.e. cell wall biogenesis^36^, auxin storage^2^, phytic acid synthesis^3^, oligosaccharides synthesis^28^ and stress tolerance^31^. In this study, we demonstrate that *myo*-inositol is involved in hook formation during skotomorphogenesis.

### Role of *myo*-inositol in hook formation

Ethylene is involved in hook formation and exogenous ethylene or its agonist results in exaggerated hook and shortening of hypocotyl length, as part of the classical triple response^42^. An increase in triple response was observed upon MI supplementation. Decrease in hook angle along with shortening of hypocotyl length was observed with increasing MI concentration. To decipher point of action of *myo*-inositol phosphate synthase in ethylene pathway, etiolated seedlings were supplemented with AgNO_3_. No hook formation was observed in etiolated seedling when treated with AgNO_3_ which is in accordance with the previous reports which suggests inhibition of the ethylene induced triple response^7^ via perturbing the ethylene perception by ETR1 and downstream signaling^58^. However, AgNO_3_ and MI combination results in hook formation in etiolated seedlings. We can therefore assume that *MIPS* acts downstream of ETR1 and results in hook formation. Up-regulation of ACO_3_ and ERF1B gene expression in etiolated seedling upon MI supplementation substantiates the hypothesis that MIPS regulates the ethylene pathway. According to previous reports, MI supplementation have no effect on MIPS expression in light grown *Arabidopsis* seedlings^33^ and it decreases MIPS expression in yeast^57^. In etiolated seedlings exogenous MI induces its own expression. As MIPS can be regulated by phosphorylation^13^, we have hypothesized that in light condition, expression of MIPS solely dependent on light however in dark its dependent on its activity which can regulated by the level of *myo*-inositol and hormone like Brassinosteroid.

### *Myo*-inositol works upstream brassinosteroid response during hook formation

Involvement of brassinosteroid in hook formation directed us to investigate the effect of *myo*-inositol phosphate synthase during brassinosteroid signaling. We checked the response of etiolated seedling grown on media supplemented with different concentrations of EBL and found, as previously reported, randomized growth of hypocotyl along with no hook formation in etiolated seedlings^22^. This effect increases with increase in concentration of EBL. However, *myo*-inositol supplementation resulted in reduced randomization or WT phenotype. Therefore, we proposed that brassinosteroid might reduce the MI level and exogenous supplementation restores it. Upon EBL treatment, enhanced randomization of hypocotyl has been reported in auxin polar transport mutant^22^ and disturbing basipetal auxin transport which result in disappearance of hook^18,19^ suggests perturbation of polar auxin transport by higher concentration brassinosteroid. Altered polar auxin transport has also been reported in *Atmips1* due to reduced level of phosphatidylinositol which affects PIN2 and PIN1 trafficking resulting in altered pattern formation^11,37^. Increase in expression of PIN3 and ABCB19 efflux carrier upon MI and decrease upon EBL supplementation, imply that randomized growth of etiolated seedlings upon EBL is due to disturbance of auxin transport via reducing the level of MI. *Atmips1* also resembles auxin mutant^37^ and as GSK-3/SHAGGY like kinases are required for optimal synthesis of *myo*-inositol^6^. The presence of GSK-3/SHAGGY like kinase i.e. BIN2, a negative regulator of brassinosteroid signaling and antagonism of *myo*-inositol on BR effect prompted us to speculate that the BIN2 might be involved in *myo*-inositol synthesis via phosphorylation of serine moiety of highly conserved motif (NGSPQN)^13,20,23,24,47^. Inactivation of BIN2 by brassinosteroid signaling might lead to decrease in *myo*-inositol synthesis in dark resulting in randomized growth which is antagonized by supplementation of MI. In support of the later, we further investigated the effect of EBL in combination of LiCl on etiolated seedling and found excessive randomization even on 100 nM EBL with 10 mM of LiCl and similar result was observed combinatorial assay with EBL and AgNO_3_. Enhanced randomization of etiolated seedling with treatment with AgNO_3_ has also been reported by Gupta et al^22^. This study indicates that the brassinosteroid signaling decreases the ethylene response via inactivating the *MIPS* and results in increase in hypocotyl growth. However, treatment with saturating concentration of EBL results in antagonism probably due to a feedback mechanism^5,14,40^.

We checked the response of etiolated seedling in combination of BRZ and MI and found hook formation in etiolated seedlings compared to etiolated seedlings grown on only BRZ which suggests the upstream role of *myo*-inositol phosphate synthase to brassinosteroid in hook formation substantiating the role of MIPS in ethylene synthesis. This indicates the existence of a cross talk between ethylene, brassinosteroid and auxin at the MIPS protein level leading to *myo*-inositol being crucial for hook as well as proper hypocotyl development.

### *Myo*-inositol phosphate synthase and Auxin Levels

Due to importance of differential auxin accumulation in hook formation, similar behavior of *Atmips1* and *Ataux1* mutant along with altered trafficking of PIN2 protein in *Atmips1*^11^ led us to investigate the relationship of *myo*-inositol and auxin during hook formation. We carried out the combinatorial assay and found agravitropic behavior of etiolated seedlings with increase in IAA concentration. However, MI supplementation resulted in distinct decrease in hook angle and gravitropic growth. Hookless phenotype is observed upon over-accumulation of the active auxin (IAA), mutation of auxin polar transporter and treatment with inhibitor of auxin polar transport^1,8^. Previous report also indicates a crosstalk between ethylene and auxin in hook formation^44^ as inhibition of hook formation occur upon NPA treatment in *eto1* and *ctr1* mutant^34^ plus the restoration of hook occur in ethylene-insensitive mutant by auxin^25^. In the present study, we observe that *myo*-inositol was able to antagonize the effect of high level of IAA at 1 μM and 10 μM concentration. We thus conclude that *myo*-inositol phosphate synthase is involved in maintaining the optimal levels of auxin in the hook region via proper localization of PIN protein and ethylene synthesis which results in differential accumulation of auxin. TIBA is an auxin transport inhibitor which perturbs the auxin efflux, therefore plants treated with TIBA show agravitropic phenotype^38^. As expected hookless phenotype was observed when seedlings were grown in dark on media supplemented with TIBA along with agravitropic etiolated seedlings. Subsequent MI supplementation could not evoke hook formation but results in shortening of hypocotyl length. This suggests that differential auxin distribution is extreme downstream of *MIPS* in hook formation^1,4,18,19^.

### Skotomorphogenesis is under control of ethylene, brassinosteroid and auxin integration via *myo*-inositol

**Figure 7:**
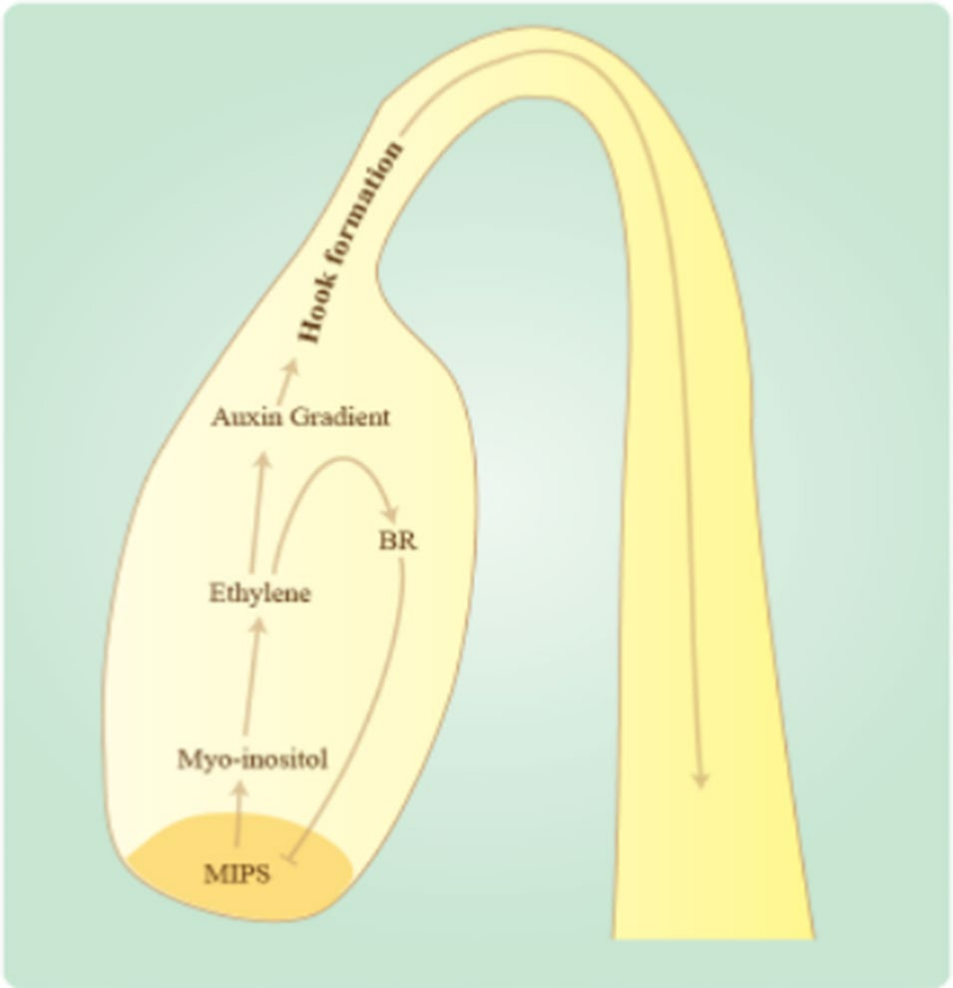
A model based on findings and published data. During hook formation, MI induces the ethylene response which induces brassinosteroid response, however this results in inhibition of MI synthesis. Differential distribution of auxin is maintained via MI through PIN protein. It is therefore MI is one of the important regulators of apical hook formation, maintenance and opening.

Hook formation and maintenance is complex interplay of ethylene, brassinosteroid and auxin. Our investigation suggests a MI as another factor involved in hook formation. Our investigation suggests MI is an important central coordinator factor involved in hook formation and hypocotyl growth. Investigation with inhibitors of ethylene, brassinosteroid and auxin on etiolated seedling in hook formation was carried out and found increase in number of seedlings showing open cotyledon in combination compared to individual composition. It could be assumed that all the three hormones along with *myo*-inositol are necessary for hook formation and their action are additive in nature as we could see additive effect of inhibitors in combinations. Another observation made was on hypocotyl length i.e. brassinosteroid is required for the hypocotyl growth as decrease in hypocotyl length was seen in BRZ treated etiolated seedlings in combination of AgNO_3_ and TIBA whereas the MI was antagonizing the effect of brassinosteroid as increase in hypocotyl length was observed in LiCl and BRZ treated etiolated seedling. Previous reports also suggest that hypocotyl growth is associated with brassinosteroid and ethylene antagonizes brassinosteroid effect in hypocotyl growth^22^. It is therefore MI is one of the important regulators of apical hook formation and hypocotyl growth. A model based on findings and published data have been proposed (Fig. 7)**.** According to which, MI induces the ethylene response which result in induction of brassinosteroid signaling and auxin gradient. Moreover, differential distribution of auxin is maintained via MI through PIN protein. Activation of brassinosteroid singling results in inhibition of MI synthesis. This will lead to inhibition of this cycle and results in opening of apical hook.

## Material and methods

### Plant sample, growth and treatments

*Arabidopsis thaliana* (Col-0) seeds were sterilized with sodium hypochlorite for 10 min and three time washed with RO water. Seedlings were grown on half strength MS media (DUCHEFA BIOCHEMIE) without sucrose supplemented different concentration of MI, EBL, IAA, ACC, LiCl, BRZ, TIBA, AgNO_3_ as specified. Seeds were then cold stratified and exposed to 12 h light stimulate uniform germination. Plates were wrapped with aluminum foil and then transferred to growth chamber for 5 days at 22 ± 1 °C.

### Hook angle measurement

Apical hook angle and hypocotyl length was measured using the ImageJ software^48^. Apical hook angle was measured by taking hypocotyl as a reference. When hook opens up, it creates a straight line. We considered it as 180° and opening of hook as an increase in hook angle. We measured the acute angle formed between the cotyledon and hypocotyl, i.e. inner edge of the apical hook. The photographs are of the 5-day old etiolated seedlings.

### Mutant confirmation

*Atmips1* (salk_02779) was confirmed using left primer (LP), right primer (RP) and T-DNA-specific primers (LBb1.3) listed in Supplementary Table S1. LP, RP and LP, LBb1.3 primer combination set was used to amplify wild type and mutant PCR product which corresponds to 1054 bp and 534–834 bp, respectively. The PCR was performed with annealing temperature of 55 °C. and PCR products were then visualized on 1% agarose gels. Primers used were listed in Supplementary Table S1.

### RNA isolation and cDNA synthesis

RNA was isolated from the different plant tissues (control and treated sample) using RNeasy plant mini kit (Qiagen, Germany). 5-day old control and treated etiolated seedlings were ground with liquid nitrogen and further proceeded according to the kit manual. In-column DNase treatment was done to remove the genomic DNA contamination. Quality and quantity of RNA samples were done by gel electrophoresis and nanodrop. 2 μg of RNA was used to make the cDNA using High-Capacity cDNA Reverse Transcription Kit (THERMOFISHER SCIENTIFIC) and SuperScript III First-Strand Synthesis System (THERMOFISHER SCIENTIFIC) for Full length cDNA synthesis. SYBR green PCR master mix (THERMOFISHER SCIENTIFIC) was used for qPCR analysis using primers listed in Supplementary Table S1.

### GUS assay

β-Glucuronidase activity in 5-days old *Arabidopsis* etiolated seedlings harboring MIPS1 promoters fused with Egfp:uidA gene were checked according to the protocol described by Jefferson et al.^26^. 5-days old Arabidopsis seedling were harvested and dipped in GUS staining buffer for 24 h at 37 °C and plant samples were then rinsed with 70% ethanol to remove chlorophyll from the stained tissue. 5-day old etiolated seedlings grown on media supplemented with MI, ACC, AgNO_3_, EBL, and BRZ were GUS stained and staining was observed using stereo microscope Leica M205 A (Leica, Germany).

### Statistical analyses

All values reported in this work are the average of at least two to three independent biological replicates having at least 15 seedlings each. Error bars represent SE. Statistical differences between control and each treatment were analyzed using Student’s t test with paired two-tailed distribution.

## Supplementary information

Supplementary Information
