# Supplementary material for: Role of myo-inositol during skotomorphogenesis in Arabidopsis
Source: Sci Rep. 2020 Oct 15;10:17329. doi: 10.1038/s41598-020-73677-x (PMC7567114; doi:10.1038/s41598-020-73677-x)
Supplement: Supplementary file 1 — Supplementary Information [file 41598_2020_73677_MOESM1_ESM.pdf]

Title: Role of Myo-inositol during Skotomorphogenesis in Arabidopsis.

Authors:
Naveen Sharma, Chanderkant Chaudhary, Paramjit Khurana*

Address and author details
Department of Plant Molecular Biology, University of Delhi South Campus, Benito Juarez Road, New Delhi, 110021, India,
*Corresponding author E-mail: param@genomeindia.org

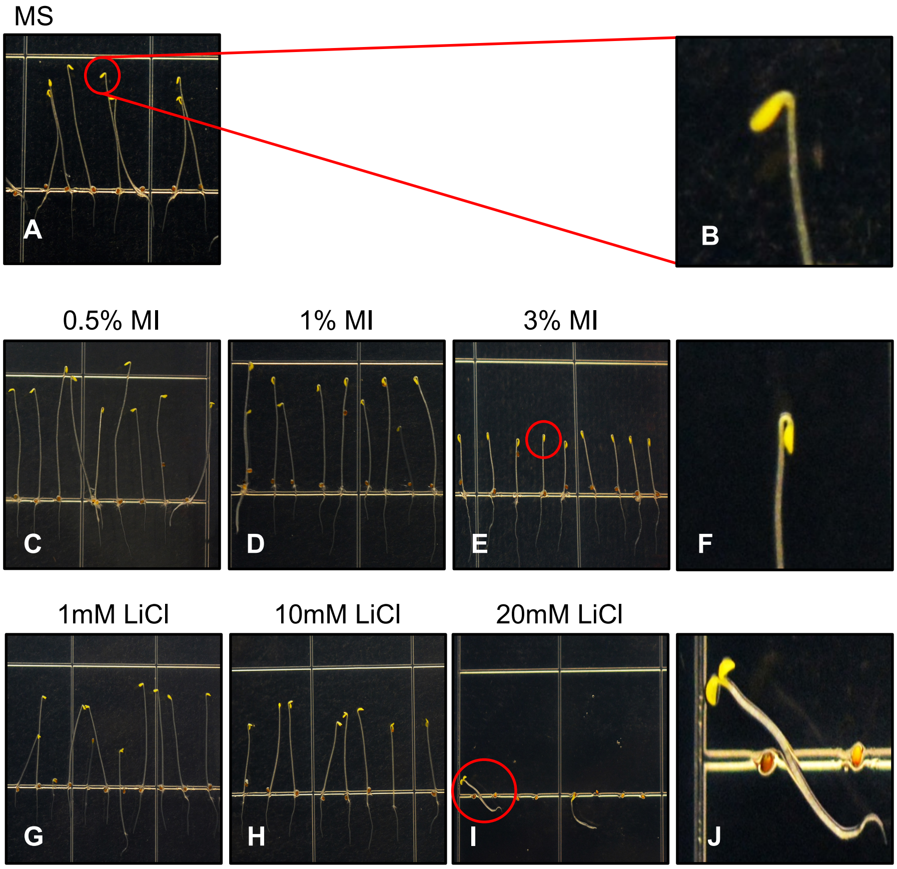

Suppl Figure 1. Responses of etiolated *Arabidopsis* seedlings to myo-inositol and LiCl. A. 5-day old etiolated seedling grown on ½ MS media without chemical treatment. C,D,E. Etiolated seedling grown on media containing 0.5%, 1%, 3% MI, respectively. G,H,I. Etiolated seedling grown on media containing 1mM, 10mM, 20mM LiCl, respectively. B,F,J. Enlarged view of selected area. Red colour circle depict the selected area.

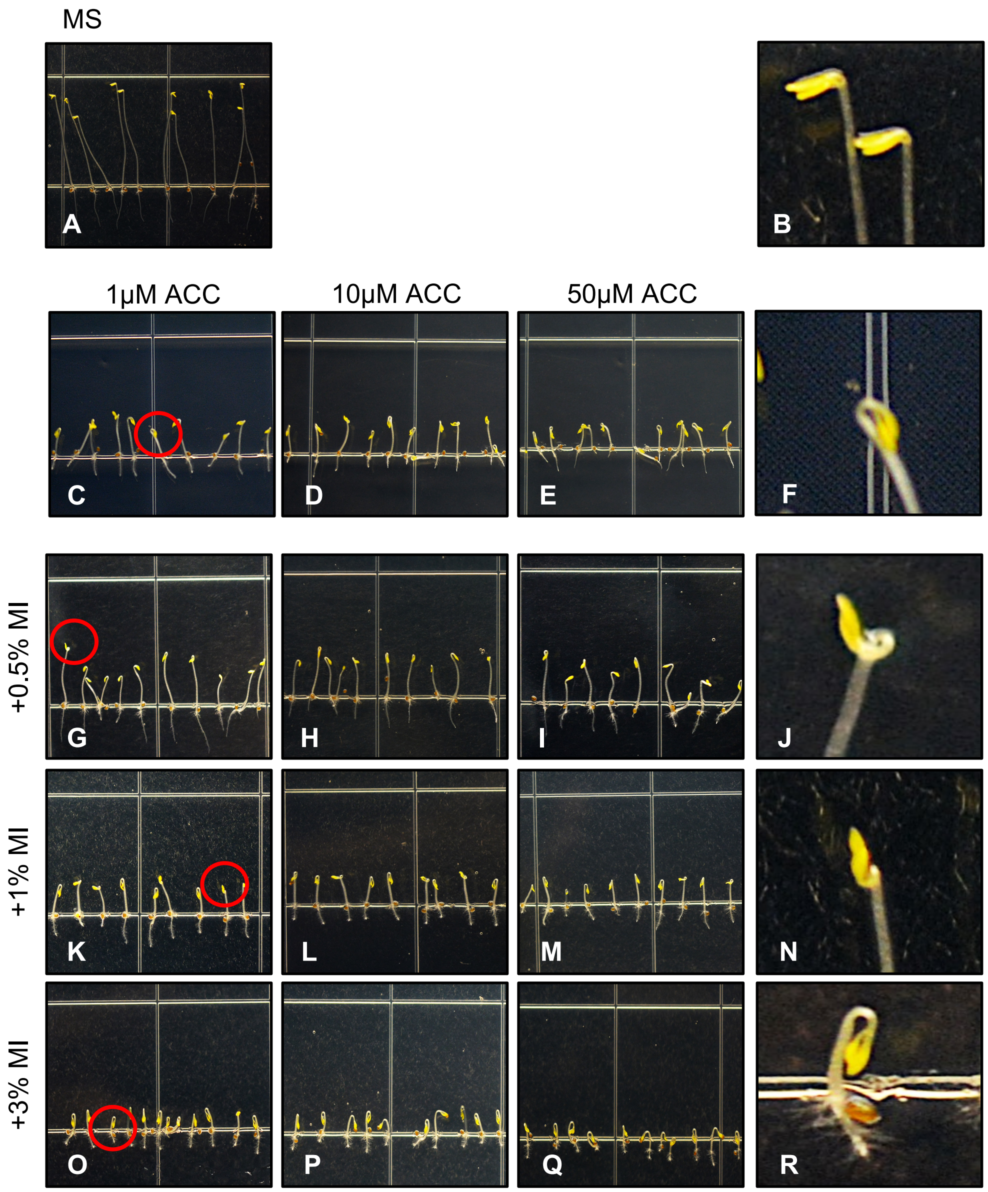

Suppl Figure 2. Responses of etiolated *Arabidopsis* seedlings to different combination of ACC and MI. A. 5-day old etiolated seedling grown on ½ MS media without chemical treatment. C,D,E. Etiolated seedling grown on media containing 1μM, 10μM, 50μM ACC, respectively. G,H,I. Etiolated seedling grown on media containing 1μM, 10μM, 50μM ACC supplemented with 0.5% MI, respectively. K,L,M. Etiolated seedling grown on media containing 1μM, 10μM, 50μM ACC supplemented with 1% MI. O,P,Q. Etiolated seedling grown on media containing 1μM, 10μM, 50μM ACC supplemented with 3% MI. B, F, J, N, R. Enlarged view of selected area. Red colour circle depict the selected area. S. Percentage of etiolated seedlings showing triple response upon different MI & ACC treatment.

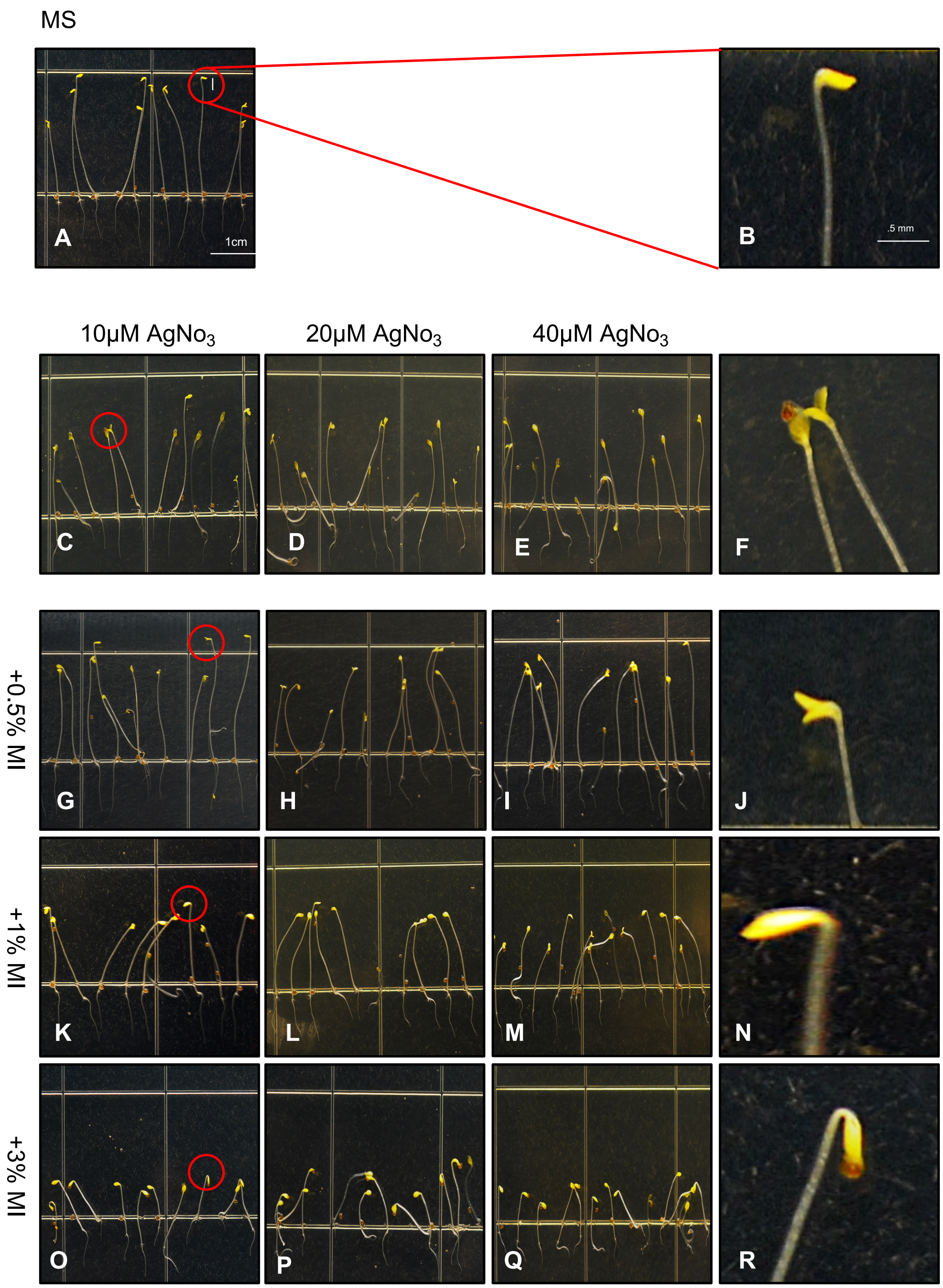

Suppl Figure 3. Responses of etiolated *Arabidopsis* seedlings to different combination of $AgNo_3$ and MI. A. 5-day old etiolated seedling grown on ½ MS media without chemical treatment. C,D,E. Etiolated seedling grown on media containing 10μM, 20μM, 40μM $AgNo_3$, respectively. G,H,I. Etiolated seedling grown on media containing 10μM, 20μM, 40μM $AgNo_3$ supplemented with 0.5% MI, respectively. K,L,M. Etiolated seedling grown on media containing 10μM, 20μM, 40μM $AgNo_3$ supplemented with 1% MI. O,P,Q. Etiolated seedling grown on media containing 10μM, 20μM, 40μM $AgNo_3$ supplemented with 3% MI. B, F, J, N, R. Enlarged view of selected area. Red colour circle depict the selected area.

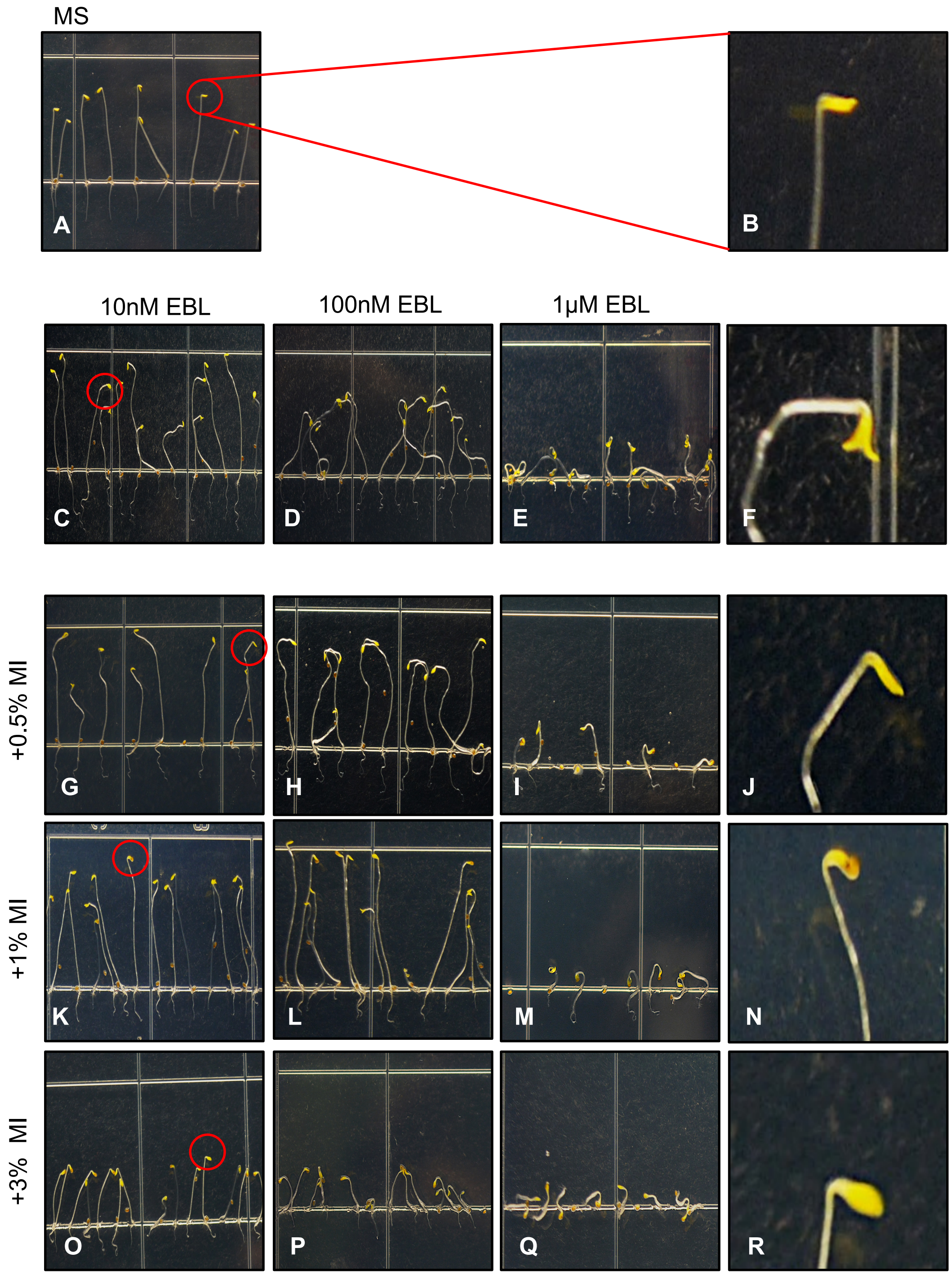

Suppl Figure 4. Responses of etiolated *Arabidopsis* seedlings to different combination of EBL and MI. A. 5-day old etiolated seedling grown on ½ MS media without chemical treatment. C,D,E. Etiolated seedling grown on media containing 10nM, 100nM, 1µM EBL, respectively. G,H,I. Etiolated seedling grown on media containing 10nM, 100nM, 1µM EBL supplemented with 0.5% MI, respectively. K,L,M. Etiolated seedling grown on media containing 10nM, 100nM, 1µM M BR supplemented with 1% MI. O,P,Q. Etiolated seedling grown on media containing 10nM, 100nM, 1µM EBL supplemented with 3% MI. B, F, J, N, R. Enlarged view of selected area. Red colour circle depict the selected area.

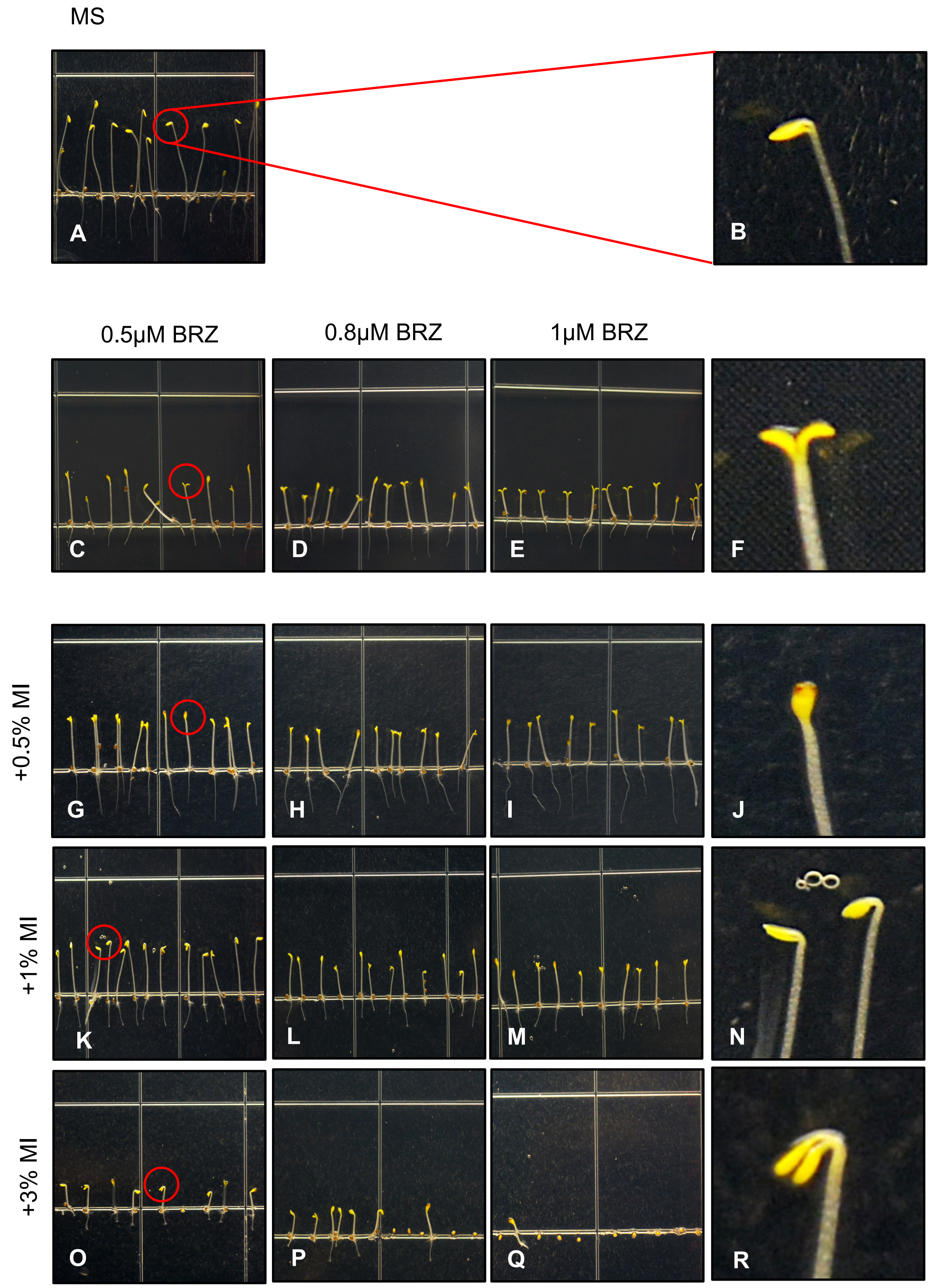

Suppl Figure 5. Responses of etiolated *Arabidopsis* seedlings to different combination of BRZ and MI. A. 5-day old etiolated seedling grown on ½ MS media without chemical treatment. C,D,E. Etiolated seedling grown on media containing 0.5μM, 0.8μM, 1μM BRZ, respectively. G,H,I. Etiolated seedling grown on media containing 0.5μM, 0.8μM, 1μM BRZ supplemented with 0.5% MI, respectively. K,L,M. Etiolated seedling grown on media containing 0.5μM, 0.8μM, 1μM BRZ supplemented with 1% MI. O,P,Q. Etiolated seedling grown on media containing 0.5μM, 0.8μM, 1μM BRZ supplemented with 3% MI. B, F, J, N, R. Enlarged view of selected area. Red colour circle depict the selected area.

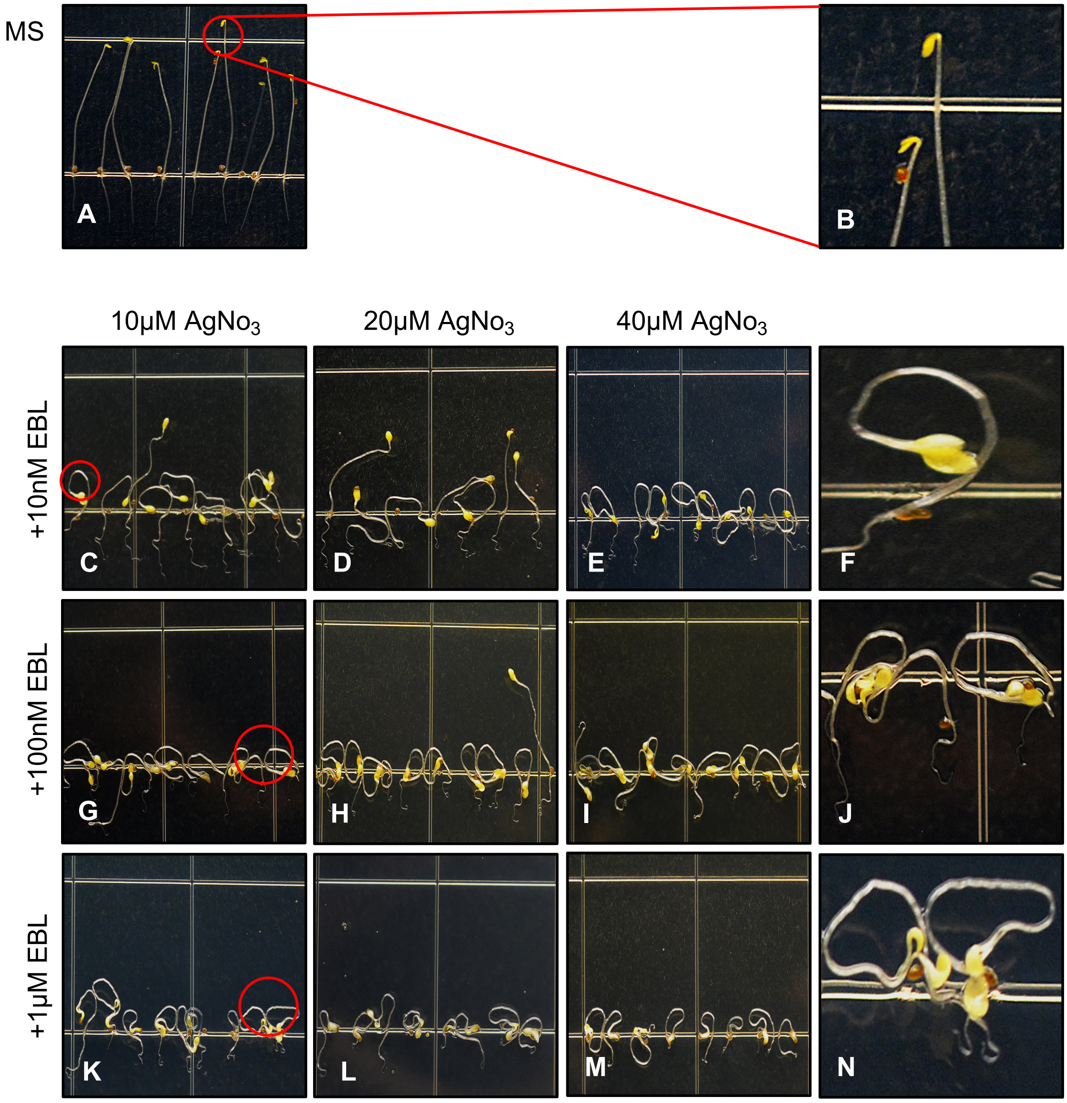

Suppl Figure 6. Responses of etiolated *Arabidopsis* seedlings to different combination of $AgNo_3$ and BR. A. 5-day old etiolated seedling grown on ½ MS media without chemical treatment. C,D,E. Etiolated seedling grown on media containing 10μM, 20μM, 40uM $AgNo_3$ supplemented with 10nM EBL, respectively. G,H,I. Etiolated seedling grown on media containing 10μM, 20μM, 40uM $AgNo_3$ supplemented with 100nM EBL. K,L,M. Etiolated seedling grown on media containing 10μM, 20μM, 40uM $AgNo_3$ supplemented with 1μM EBL. B, F, J, N. Enlarged view of selected area. Red Colour circle depict the selected area.

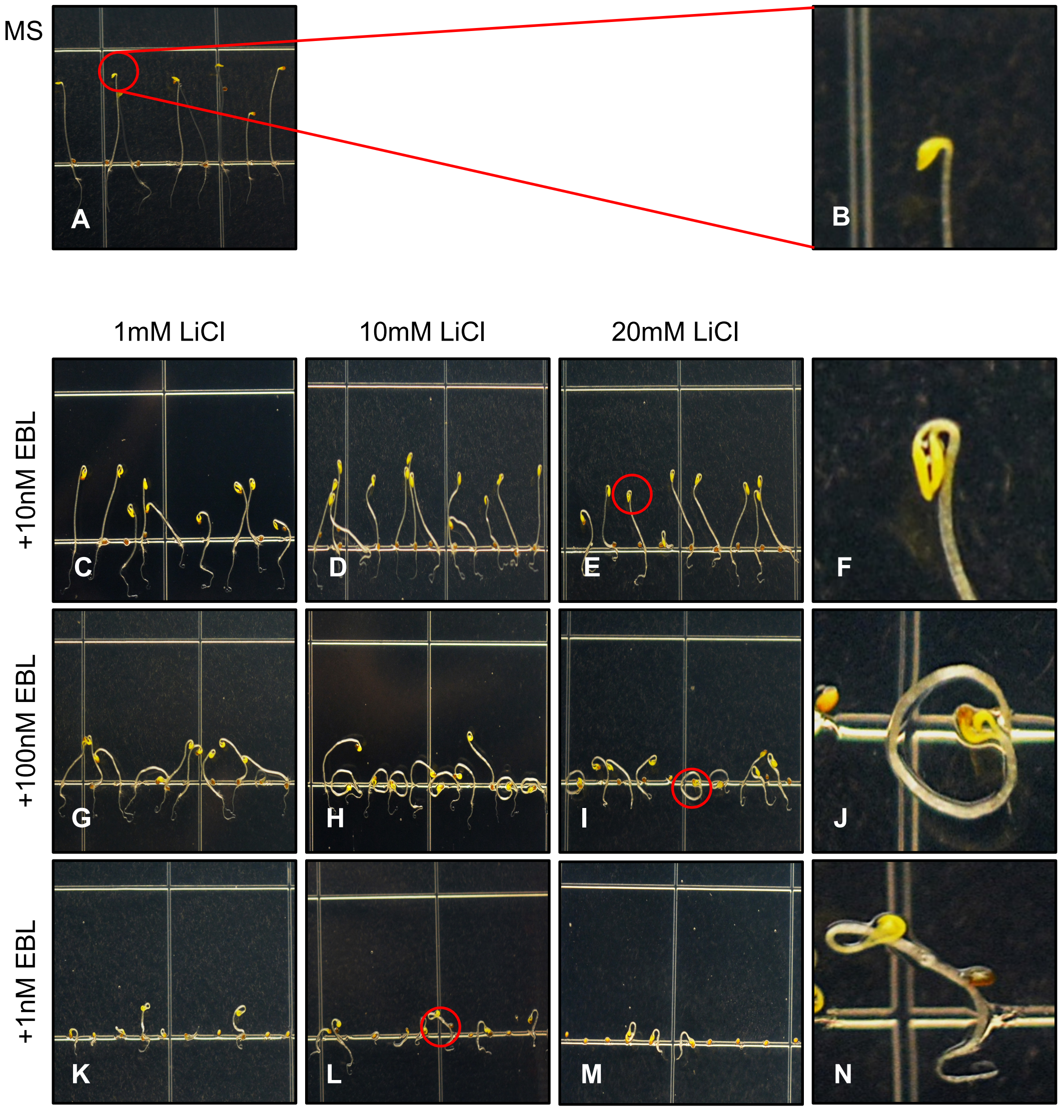

Suppl Figure 7. Responses of etiolated *Arabidopsis* seedlings to different combination of LiCl and EBL. A. 5-day old etiolated seedling grown on ½ MS media without chemical treatment. C,D,E. Etiolated seedling grown on media containing 1mM $LiCl_2$, 10mM LiCl, 20mM LiCl supplemented with 10nM EBL, respectively. G,H,I. Etiolated seedling grown on media containing 1mM, 10mM, 20mM supplemented with 100nM EBL. K,L,M. Etiolated seedling grown on media containing 1mM, 10mM, 20mM supplemented with 1µM EBL. B, F, J, N. Enlarged view of selected area. Red Colour circle depict the selected area.

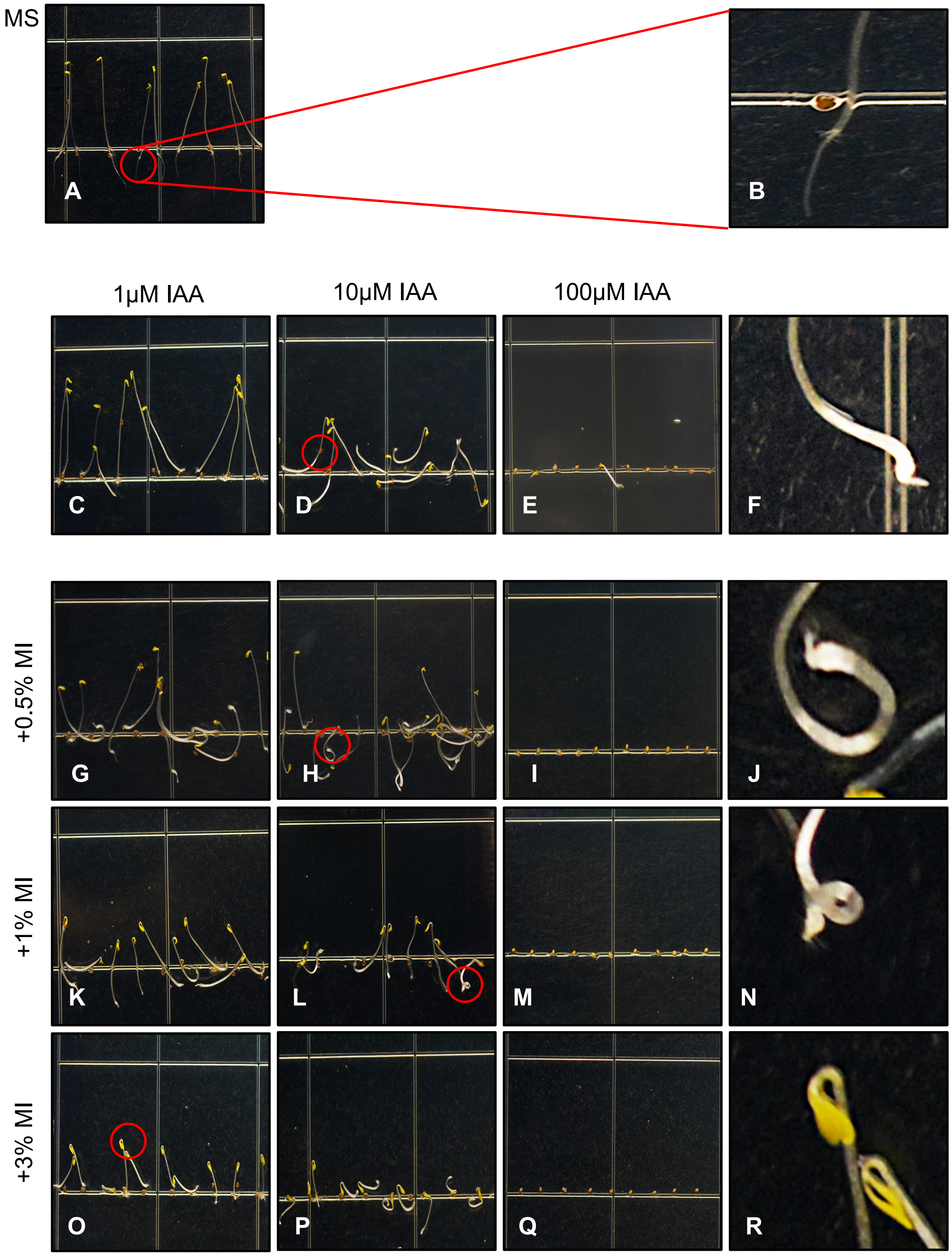

Suppl Figure 8. Responses of etiolated *Arabidopsis* seedlings to different combination of IAA and MI. A. 5-day old etiolated seedling grown on ½ MS media without chemical treatment. C, D, E. Etiolated seedling grown on media containing 1μM, 10μM, 100μM IAA supplemented with 0.5% MI, respectively. G, H, I. Etiolated seedling grown on media containing 1μM, 10μM, 100μM IAA supplemented with 1% MI. K,L,M. Etiolated seedling grown on media containing 1μM, 10μM, 100μM IAA supplemented with 3% MI. B, F, J, N, R. Enlarged view of selected area. Red Colour circle depicts the selected area.

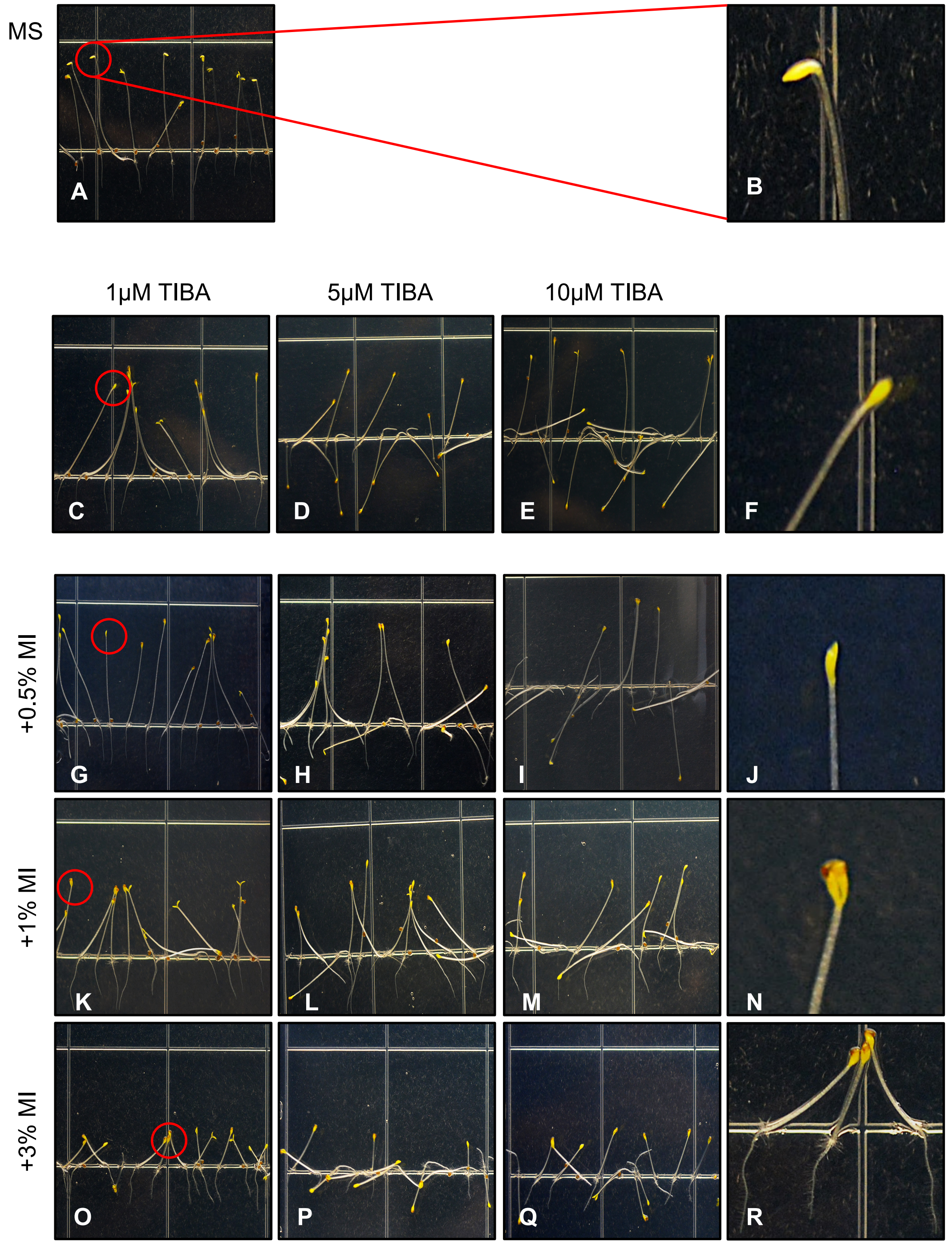

Suppl Figure 9. Responses of etiolated *Arabidopsis* seedlings to different combination of TIBA and MI. A. 5-day old etiolated seedling grown on ½ MS media without chemical treatment. C,D,E. Etiolated seedling grown on media containing 1μM, 5μM, 10μM TIBA, respectively. G,H,I. Etiolated seedling grown on media containing 1μM, 5μM, 10μM TIBA supplemented with 0.5% MI, respectively. K,L,M. Etiolated seedling grown on media containing 1μM, 5μM, 10μM TIBA supplemented with 1% MI. O,P,Q. Etiolated seedling grown on media containing 1μM, 5μM, 10μM TIBA supplemented with 3% MI. B, F, J, N, R. Enlarged view of selected area. Red colour circle depict the selected area.

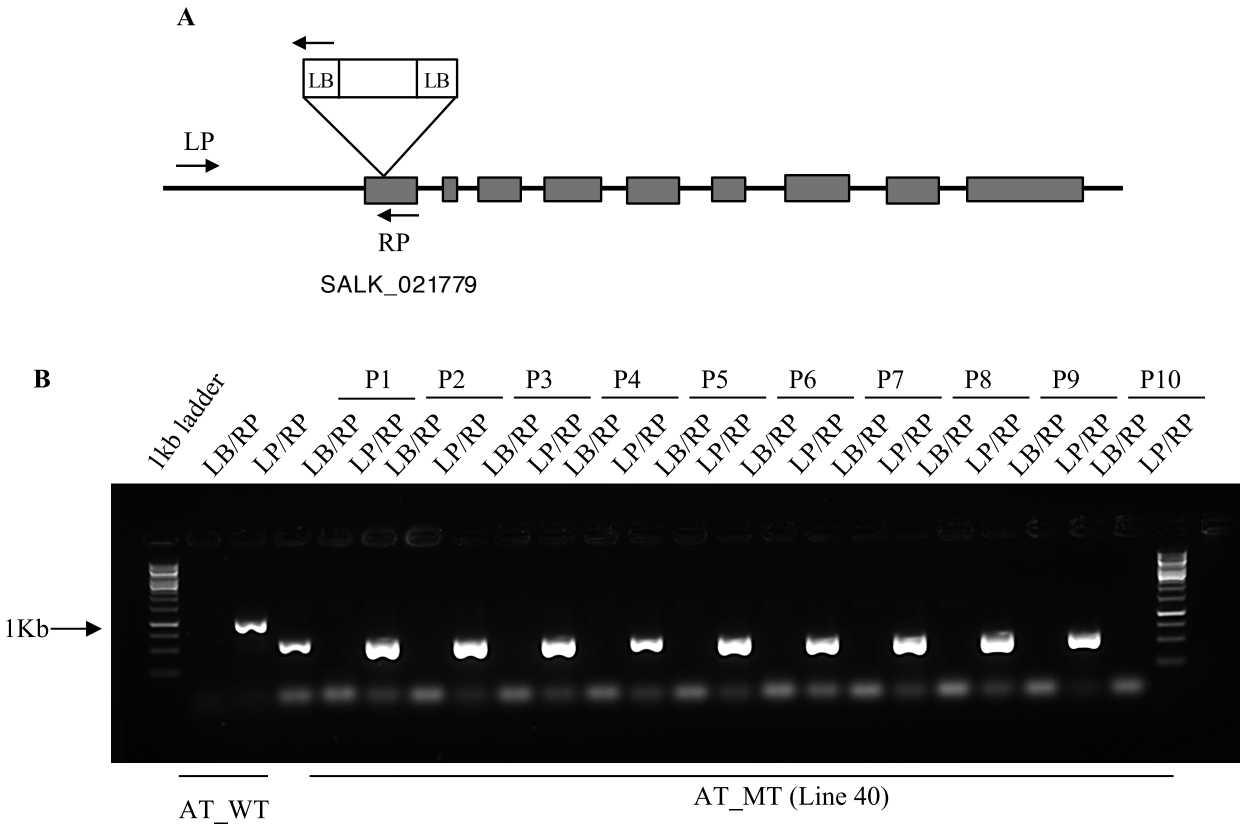

Suppl Figure 10. *Atmips1* mutant characterization. A. Schematic of T-DNA insertion sites in the *mips1* mutants (SALK_021779). Exons are shown as dark-gray boxes; the black arrows indicate primers used to amplify left border (LB) of the T-DNA; black arrows indicate the positions of gene-specific primers. B. PCR confirmation of T-DNA insertion in *mips1* mutants (SALK_021779) line 40.

Suppl Table 1. Primer list.

S No	Primer Name	
1	*Atmips1_* LP	TTCGAGGCTTAGTTGTTGGAG
2	*Atmips1_* RP	CTTTTTGGCAGGGAAAGTTTC
3	LBb1.3	ATTTTGCCGATTTCGGAAC
4	*AtMIPS1_* FP	CAAATCCGAGGGAGAGGGAAAGTTTC
5	*AtMIPS1_* RP	TGCGTTAATCACCGGAGTTCCC
6	*AtBRI1_* FP	ACCAATTGGAAGCTGACTGG
7	*AtBRI1_* RP	CCTGAAGAAGATCCGCAAAC
8	*AtBIN2_* FP	ACCCGAGCTCATATTTGGTG
9	*AtBIN2_* RP	TTCAACGAGCTGATCCACAG
10	*AtDET2_* FP	CGCCGATAAAACCTTCTTCC
11	*AtDET2_* RP	AAGCAATCGGTGGAGATACG
12	*AtBSL2_* FP	ATGGCGTAAGTTGGAACCAG
13	*AtBSL2_* RP	ACTGTTTGCATCCCTTCCAC
14	*AtACO3_* FP	GGTGATTGGGTTGATGTTCC
15	*AtACO3_* RP	AAACGACGCGATAGACATCC
16	*AtCTR1_* FP	TCAGGTTTGGGCTTGATAGG
17	*AtCTR1_* RP	CCGCAGAGGAGAAGAAATTG
18	*AtERF1B_* FP	TTCCCTTCAACGAGAACGAC
19	*AtERF1B_* RP	TAGGTTTGTTGCGTGGACTG
20	*AtSAUR15_* FP	AAGGGAATCATCGTCGACAC
21	*AtSAUR15_* RP	AAGTATGAAACCGGCACCAC
22	*AtPIN4_* FP	CAAGTCAAACAGCCATGACG
23	*AtPIN4_* RP	TTGATCGTTGTCAGGTGCTC
24	*AtABCB19_* FP	AACCGATGCCAAGACTGTTC
25	*AtABCB19_* RP	ATGGCACCAAGAGAACCAAC
